# An ontogenic study of the behavioral effects of chronic intermittent exposure to ayahuasca in mice

**DOI:** 10.1590/1414-431X20176036

**Published:** 2017-06-05

**Authors:** N.F. Correa-Netto, M.Y. Masukawa, F. Nishide, G.S. Galfano, F. Tamura, M.K. Shimizo, M.P. Marcato, J.G. Santos, A. Linardi

**Affiliations:** Departamento de Ciências Fisiológicas, Faculdade de Ciências Médicas, Santa Casa de São Paulo, São Paulo, SP, Brasil

**Keywords:** Adolescent, Anxiety, Banisteriopsis, *N,N*-Dimethyltryptamine, Infant, Memory

## Abstract

Ayahuasca is a beverage obtained from decoctions of the *Banisteriopsis caapi* plus *Psychotria viridis*. In religious contexts, ayahuasca is used by different age groups. However, little is known of the effects of ayahuasca during ontogenic development, particularly with regard to the functional characteristics of the central nervous system. Animal models are useful for studying the ontogenic effects of ayahuasca because they allow exclusion of the behavioral influence associated with the ritualistic use. We investigated the effects of exposure to ayahuasca (1.5 mL/kg, orally, twice a week) on memory and anxiety in C57BL/6 mice, with the post-natal day (PND) being used as the ontogenic criterion for classification: childhood (PND21 to PND35), adolescence (PND35 to PND63), adulthood (PND90-PND118), childhood-adolescence (PND21 to PND63), childhood-adulthood (PND21 to PND118) and adolescence-adulthood (PND35 to PND118). One day after the last ayahuasca exposure, the mice were subjected to the Morris water maze (MWM), open field and elevated plus maze tasks (EPM). Ayahuasca did not affect locomotion in the open field or open arms exploration in the EPM, but increased the risk assessment behavior in the childhood group. Ayahuasca did not cause any change in acquisition of spatial reference memory in the MWM task, but decreased the time spent on the platform quadrant during the test session in the adolescence group. These results suggest that, in mice, exposure to ayahuasca in childhood and adolescence promoted anxiety and memory impairment, respectively. However, these behavioral changes were not long-lasting since they were not observed in the childhood-adulthood and adolescence-adulthood groups.

## Introduction

The Quechua term ayahuasca, common in Peru, Bolivia, Brazil and parts of Ecuador, refers to the decoctions of the liana *Banisteriopsis caapi* with the leaves of *Psychotria viridis* that yield a thick, brown liquid (1,2). *Banisteriopsis caapi* contains the alkaloids harmine, harmaline and tetrahydroharmine, all of which belong to the β-carboline group. The leaves of *P. viridis* contain the alkaloid *N,N*-dimethyltryptamine (DMT) that is responsible for the hallucinogenic effects of ayahuasca. Harmine, tetrahydroharmine and harmaline are potent inhibitors of peripheral type-A monoamine oxidase (MAO-A). MAO inhibition in the gastrointestinal tract increases DMT bioavailability in the central nervous system (3–6). DMT is structurally similar to serotonin and its central effects result from agonistic activity at serotonergic 5-HT_1A_, 5-HT_2A_, and 5-HT_2C_ receptors that induce a hallucinogenic response (7,8).

Several ancient indigenous and mestizo traditions have used ayahuasca and the term "ayahuasca religions" commonly refers to cults that have arisen in Brazil. The second half of the 20th century witnessed the emergence of organized urban and non-indigenous religious groups that use this psychoactive beverage as part of their rituals (9,10). The most commonly recognized sacramental use of ayahuasca is among members of three churches in Brazil: União do Vegetal, Santo Daime and Barquinha. Additionally, the religious use of ayahuasca was recognized as a legal practice in Brazil by the Conselho Nacional de Políticas sobre Drogas (CONAD, or National Council on Drug Policies), in Resolution No. 5 (November 4, 2004), and its control has been guided by the publication of Resolution No. 1 (January 25, 2010) (11).

The safety of ayahuasca in regular users is an important issue. A double-blind, randomized, crossover clinical trial showed that an oral dose of encapsulated freeze-dried ayahuasca had moderate sympathomimetic effects, increased cortisol and prolactin levels, and altered the cell-mediated immunity in a group of 10 healthy volunteers (12). However, these effects were reversible in less than 24 h, suggesting that a single dose of ayahuasca can be safely administered to healthy individuals. In another study, polysomnographic analysis has shown that a single dose of ayahuasca equivalent to 1 mg DMT/kg body weight decreases the duration of rapid eye movement (REM) sleep, with a trend towards an increase in latency onset. However, ayahuasca did not induce any subjectively perceived deterioration of sleep quality or polysomnographic measured disruptions of sleep initiation or maintenance (13). Furthermore, Grob et al. (4) found no evidence of mental impairment in 26-48 year-old chronic users of ayahuasca and a more recent review of the emotional, cognitive and physical health of ayahuasca users showed that the beverage was safe and beneficial under certain conditions (14). In relation to adolescents, the consumption of ayahuasca within a religious context was not significantly associated with problems related to anxiety, body dimorphism and attention problems when compared to matched controls who did not use ayahuasca (15). These adolescents also did not differ from the control group with regard to speeded attention, visual searching, sequencing, psychomotor speed, verbal and visual abilities, memory, and mental flexibility. Indeed, both groups showed similar results in most neuropsychological measures, except in a subset of a verbal learning task (16,17). However, mean raw scores of both groups in this subset of tests did not differ from adolescent normative data. Finally, *in vitro* studies have shown that β-carbolines, including harmine and harmaline, have antimutagenic, antigenotoxic and antioxidant effects (18,19).

Apart from adults, members of ayahuasca religious groups include pregnant women, infants and adolescents, with administration of the beverage also extending to newborns, usually given a few drops at baptism, and may continue throughout childhood, depending on the parents' desire (20). Therefore, it is important to define possible differences in the behavioral effects of ayahuasca in distinct lifetime periods, primarily through ontogenic studies, i.e., the study of an organism from the embryonic phase until the adult phase that includes the different developmental stages (21,22). However, in humans, such studies are complicated by the difficulty of separating the pharmacological effects of the beverage from the potential influence of its ritualistic context.

In view of the uncertainty regarding the potential ontogenic effects of ayahuasca, in this study we investigated the effects of chronic intermittent exposure to this beverage on memory and anxiety in mice of different ages. To mimic the intermittent ingestion of ayahuasca in ritualistic use (the beverage is not ingested daily) with frequencies ranging from twice a month to several times per week (4,23), the mice were exposed to ayahuasca twice a week.

## Material and Methods

### Subjects

One hundred and twenty male C57BL/6 mice were obtained from the Center for the Development of Animal Models in Biology and Medicine at the Universidade Federal de São Paulo (CEDEME/UNIFESP, São Paulo, SP, Brazil). The mice were housed 4-6 per propylene cage (40×34×17 cm) with woodchip bedding, at 20-22°C and 50% humidity on a 12-h light/dark cycle (lights on at 7:00 am), with free access to mouse chow pellets and tap water. The cages were cleaned twice a week. The experimental protocols were approved by an institutional Committee for Ethics in Animal Use (CEUA/ISMSCSP, protocol No. 002/14) and the general ethical guidelines for animal use established by the Brazilian Society of Laboratory Animal Science (SBCAL). Brazilian legislation (Federal law No. 11,794, of October 8, 2008) and EU Directive 2010/63/EU were followed, in conjunction with the guidelines for animal experiments established by the Brazilian National Council for Animal Experimentation (CONCEA, http://www.mct.gov.br/index.php/content/view/310553.html; http://www.mct.gov.br/upd_blob/0234/234054.pdf).

### Ayahuasca

Ayahuasca was kindly provided by the Centro de Desenvolvimento Integrado Luz do Vegetal (Araçariguama, SP, Brazil). To prepare the beverage, the liana *B. caapi* was carefully washed in water and pounded with wooden mallets, whereas the leaves of *P. viridis* were simply rinsed with water. The plant material was boiled and concentrated over several hours to produce approximately 100 L of beverage. The ayahuasca extract used in the present study was the same used in a previous study by our group (24). The concentrations of psychoactive alkaloids detected by HPLC-DAD were: 2070 µg/mL N,N-dimethyltryptamine, 147.5 µg/mL harmaline, 2894 µg/mL harmine and 1893 µg/mL tetrahydroharmine.

### Treatment

Ayahuasca was administered orally at a dose of 1.5 mL/kg. This dose was based on the volume used by humans in religious rituals, which is approximately 100 mL/70 kg (25,26) and was also considered as a typical dose in a previous study in Wistar rats (27). The mice received the beverage twice a week by gavage since, as indicated in the Introduction, ayahuasca use in the ritualistic context does not occur daily, but intermittently (4,28,29). The control group received water in a similar procedure. The mice were grouped based on their post-natal day (PND) which was used as the ontogenic parameter (Table 1). One day after the last ayahuasca exposure, the mice were subjected to the Morris water maze (MWM), open field and elevated plus maze (EPM) tasks.

**Table 1.** Experimental groups.

Group (n=8-12)	Treatment period	Total number of ayahuasca or water administrations
Aya-Child or Cont-Child	PND21-PND35	4
Aya-Adol or Cont-Adol	PND35-PND62	8
Aya-Adul or Cont-Adult	PND90-PND118	8
Aya-Child-Adol or Cont-Child-Adol	PND21-PND62	10
Aya-Child-Adult or Cont-Child-Adult	PND21-PND118	28
Aya-Adol-Adult or Cont-Adol-Adult	PND35-PND118	24

Mice received ayahuasca or water (1.5 mL/kg each, orally) twice a week. The post-natal day (PND) was used as the ontogenic parameter. Child: childhood; Adol: adolescence; Aya: mice treated with ayahuasca; Cont: mice treated with water.

### Behavioral tests

All behavioral tests were done between 10:00 am and 3:00 pm in a sound-proof room, 24 h after the last administration of the beverage. The tests were run for 7 days (D1, D2, D3, D4, D5, D6, and D7) after treatment. The open field and EPM tests were run on D1 (in the morning and afternoon, respectively) and the MWM test was done from D2 to D7 (in the morning). The performances of the mice were recorded in all tests and the data were analyzed with an image capture system (SMART 2.1, Panlab, Spain).

### Open field test

The open field test was used to assess locomotor activity, an important indicator of drug toxicity, and also to rule out any confounding effect on the learning and memory test (MWM). This test allowed us to monitor habituation in rodents (30). The mice were placed individually in an acrylic cage (23×42×30 cm) and the distance covered in 30 min was measured.

### EPM test

The EPM test is a paradigm for assessing anxiety since it is based on the natural aversion of rodents to open and elevated areas, generally preferring dark, closed areas. Hence, the relative exploration in the open arms compared to both open and closed arms indicates the level of anxiety (31). The apparatus consisted of four arms (30×5 cm) elevated 40 cm above floor level and connected to each other by a central platform (5×5 cm). Two of the arms were closed with 18-cm high walls and the other two arms were open.

Each mouse was placed in the center section and left to explore the maze for a single 5 min session. The number of entries and time spent in the open and closed arms were measured. These variables allowed us to determine the percentage of exploration (entries and time spent) in the open arms in relation to both open and closed arms. The number of entries into the closed arms was also used as an indicator of general locomotor activity (32).

Several studies have suggested that more detailed behavioral analyses improve the validity of the EPM (31,32). To complement our findings, we measured the time spent with risk assessment, a stretched-attend posture directed to open and closed arms in which the animal performs a forward elongation of the neck and shoulders followed by retraction to the original posture (33). The risk assessment behavior was estimated by observing the movement of the mouse from the closed arms to the center of the platform and from there to the open arms.

### MWM test

The test was adapted from Morris et al. (34). The apparatus consists of a circular water pool 150 cm in diameter containing 8 cm deep water. In the acquisition phase (D2 to D6), the mouse was required to find a hidden platform positioned at the same place in all trials of this phase (the platform was submerged 2 cm below the water surface). Each mouse underwent four acquisition trials per day for 5 consecutive days. A trial was initiated by lowering the mouse into the water by its tail while facing the side wall of the pool at a predetermined position. The trial ended when the mouse found the platform or after 120 s had elapsed. If the mouse failed to find the platform within 120 s, it was gently guided to the platform and was allowed to remain there for 30 s. At the end of each trial, the mouse was dried and kept warm in a heated box for 15 min before the next trial. The results are reported as the mean of the four daily trials. The means obtained on the 5 consecutive days allowed us to define a learning curve (acquisition of spatial reference memory). One day after the acquisition phase (D7), the mice underwent the probe trial to assess memory retrieval. The hidden platform was removed from the water pool and the mice were allowed to swim for 60 s. The time spent and distance moved in the platform quadrant, and the number of crossings that coincided with the exact position of the platform, were registered.

### Statistical analysis

Quantitative data are reported as means±SE. The extent of locomotion in the open field, the EPM parameters (percentage of entries and time spent in the open arms, and in risk assessment) and the probe trial of the MWM test (time spent and the number of crossings over the platform position) were analyzed using Student's unpaired *t*-test. One-way ANOVA for repeated measures was used to analyze the acquisition phase of the MWM test (latency in finding the hidden platform during the 5 days). The Newman Keuls *post-hoc* analysis was used when ANOVA detected significant differences among experimental groups.

The normality of the data analyzed in the Student's unpaired *t*-test and one-way ANOVA for repeated measures test was verified by Shapiro-Wilk's test. In addition, the Levene test for homogeneity of variance and the Mauchly sphericity test were applied for data analyzed by one-way ANOVA.

The level of significance was set at P<0.05. All data analyses were done using SPSS statistics v.21 software (IBM Corp., USA).

## Results

### Locomotor activity in the open field

The Shapiro-Wilk normality test showed no significant effects indicating the normal distribution of the data. Likewise, Levene's test and the Mauchly's sphericity test did not find significant differences in the data analyzed by one-way ANOVA. It is important to note that a P<0.05 indicates non-homogeneity or non-sphericity of the data.

**Figure 1.**
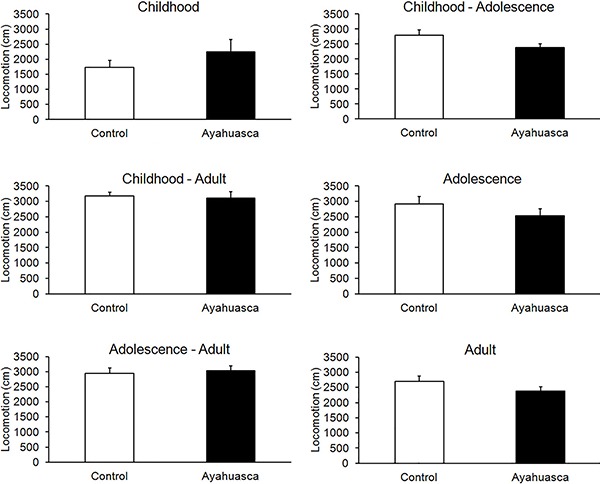
Locomotor activity of mice in the open field test. Control mice received water whereas treated mice received ayahuasca (1.5 mL/kg, orally, for treatment and control) in the different periods of development. Locomotor activity was monitored for 5 min. Data are reported as the mean±SE (n=8-12/group).

Figure 1 shows the locomotor activity in the open field. There was no significant difference between the control and ayahuasca-treated mice in any of the age groups or their combinations, as assessed by Student's *t*-test: childhood [t(19)=1.1527; P=0.2633], adolescence [t(20)=-1.1326; P=0.2707], adult [t(17)=-1.4845; P=0.1559], childhood-adolescence [t(15)=-1.8456; P=0.0847], childhood-adult [t(14)=-0.2520; P=0.8046] and adolescent-adult [t(17)=0.4359; P=0.6683] groups.

### EPM test

**Figure 2.**
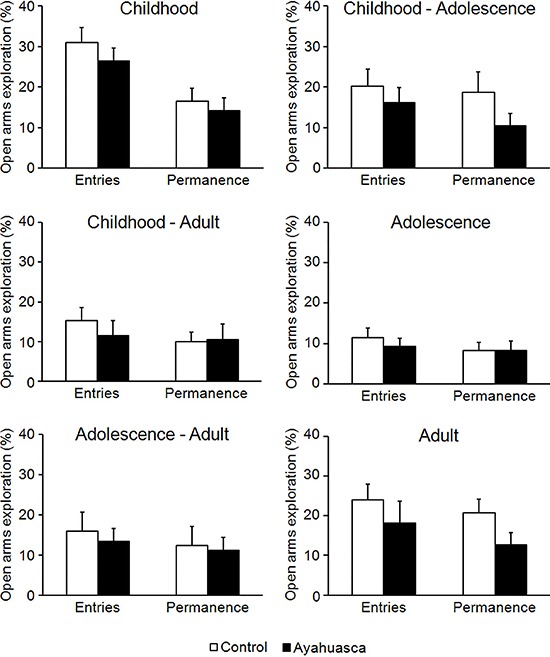
Open arms exploration (percentage of entries and of time spent in open arms) in the elevated plus maze (EPM) test. Control mice received water whereas treated mice received ayahuasca (1.5 mL/kg, orally, in both cases) in the different periods of development. Locomotor activity was monitored for 5 min. Data are reported as the mean±SE (n=8-12/group).

Figure 2 shows the open arms exploration parameters in the EPM test. Treatment with ayahuasca did not significantly affect the percentage of entries into open arms within the different age groups when compared to control mice (Student's *t*-test): childhood [t(19)=0.7619; P=0.4554], adolescence [t(20)=0.6443; P=0.5266], adult [t(21)=1.1377; P=0.2680], childhood-adolescence [t(15)=0.6263; P=0.5404], childhood-adult [t(14)=0.7492; P=0.4661] and adolescent-adult [t(17)=0.4711; P=0.6436] groups. As with the percentage of entries, there was no difference in the length of stay (reported as a percentage) in the open arms between control and ayahuasca-treated mice, as assessed by Student's *t*-test: childhood [t(19)=0.5717; P=0.5742], adolescence [t(20)=-0.0052; P=0.9958], adult [t(21)=1.3007; P=0.2074], childhood-adolescence [t(15)=1.3527; P=0.1962], childhood-adult [t(14)=-0.0887; P=0.9305] and adolescent-adult [t(17)=0.2279; P=0.8229] groups.

**Figure 3.**
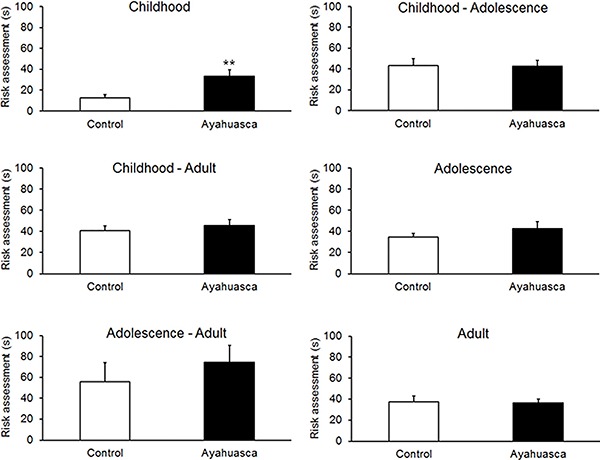
Time spent by mice in risk assessment behavior in the elevated plus maze (EPM) test. Control mice received water whereas treated mice received ayahuasca (1.5 mL/kg, orally, in both cases) in the different periods of development. Data are reported as the mean±SE (n=8-12/group). **P<0.01 compared to the respective control group (Student's unpaired *t*-test).

The risk assessment behavior in the EPM test is shown in Figure 3. Mice treated with ayahuasca in childhood spent more time in risk assessment than control mice [t(19)=-3.2684; P=0.0040; Student's *t*-test], but there was no significant difference in the other age groups: adolescence [t(20)=-1.0618; P=0.3009], adult [t(21)=0.1867; P=0.8536], childhood-adolescence [t(15)=0.0961; P=0.9246], childhood-adult [t(14)=-0.6553; P=0.5228] and adolescent-adult [t(17)=-0.7661; P=0.4541] groups.

### MWM test

**Figure 4.**
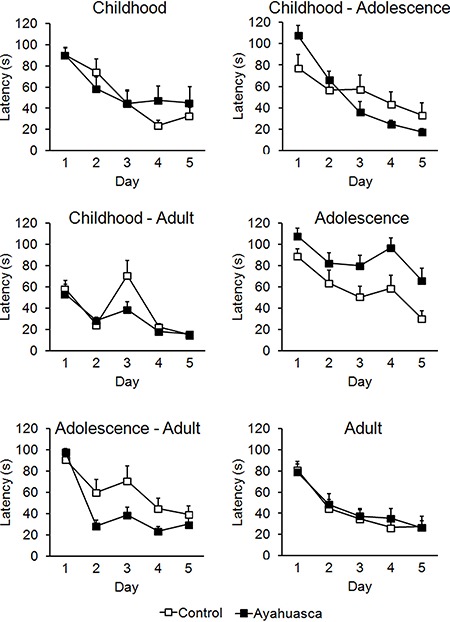
Mean latency during the acquisition phase of the Morris water maze (MWM) test. The latency to find the hidden platform was measured during 5 days of the acquisition phase. Control mice received water whereas treated mice received ayahuasca (1.5 mL/kg, orally, in both cases) in the different periods of development. Data are reported as the mean±SE (n=8-12/group). One way ANOVA for repeated measures and Newman Keuls post hoc.

*Spatial reference memory acquisition*. Figure 4 and Table 2 show that overall there was a decrease in the latency to find the platform in the 5 days of the acquisition phase in control and ayahuasca-treated mice of different age groups.

**Table 2.** Statistical analysis of spatial reference memory acquisition in the MWM test.

Group	Time effect	Treatment effect	Time × treatment interaction
Child	Yes [F(4,76)=24.4810; P=0.0011]	No [F(1,19)=0.1061; P=0.7842]	Yes [F(4,76)=2.5764; P=0.0441]
Adol	Yes [F(4,80)=12.4341; P=0.0012]	Yes [F(1,20)=7.1250; P=0.0147]	No [F(4,80)=0.7733; P=0.5458]
Adult	Yes [F(4,84)=24.4101; P=0.0013]	No [F(1,21)=0.0606; P=0.8079]	No [F(4,84)=0.2443; P=0.9124]
Child-Adol	Yes [F(4,60)=33.4881; P=0.0015]	No [F(1,15)=0.0805; P=0.7805]	Yes [F(4,60)=6.1132; P=0.0012]
Child-Adult	Yes [F(4,56)=20.8271; P=0.0013]	No [F(1,14)=0.0066; P=0.9365]	No [F(4,56)=0.3225; P=0.8617]
Adol-Adult	Yes [F(4,68)=29.9341; P=0.0012]	Yes [F(1,17)=4.5188; P=0.0485]	Yes [F(4,68)=3.2988; P=0.0157]

One-way ANOVA for repeated measures was used to analyze the acquisition phase of the Morris water maze (MWM) test (latency in finding the hidden platform during the 5 days). The Newman-Keuls *post-hoc* test was used when ANOVA detected significant differences among experimental groups. Child: childhood; Adol: adolescence.

For the childhood group, a significant effect of interaction between factors was found. The decrease in the latency to find the platform was faster and stronger in ayahuasca-treated mice than in the control group.

For the adolescence group, a significant effect of treatment was found, but there was no interaction between factors. Although the learning curves were similar, all of the latencies were longer in ayahuasca-treated mice than in the control group.

For the childhood-adolescence group, a significant effect of interaction between factors was found. The decrease in the latency to find the platform was faster and stronger in ayahuasca-treated mice than in the control group.

For the adolescence-adult group, a significant effect of treatment was found and there was interaction between factors. The decrease in the latency to find the platform was faster and stronger in ayahuasca-treated mice than in the control group.

There was no significant effect of treatment in the childhood-adult and adult groups.

**Figure 5.**
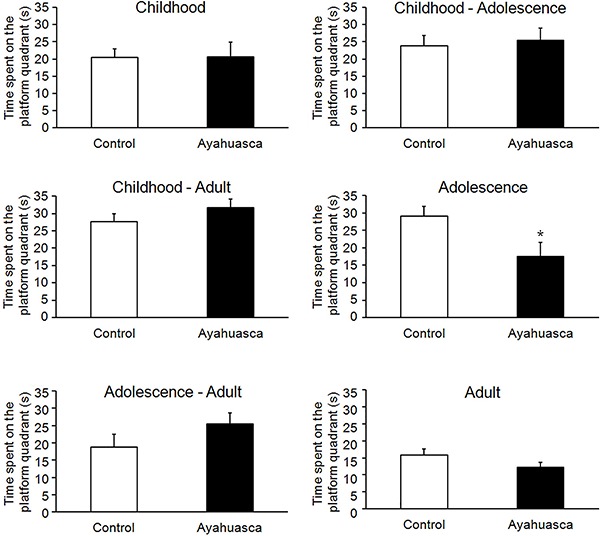
Time spent in the platform quadrant during the probe trial session. Control mice received water whereas treated mice received ayahuasca (1.5 mL/kg, orally, in both cases) in the different periods of development. Data are reported as the mean±SE (n=8-12/group). *P<0.05 compared to the corresponding control group (Student's unpaired *t*-test).

**Figure 6.**
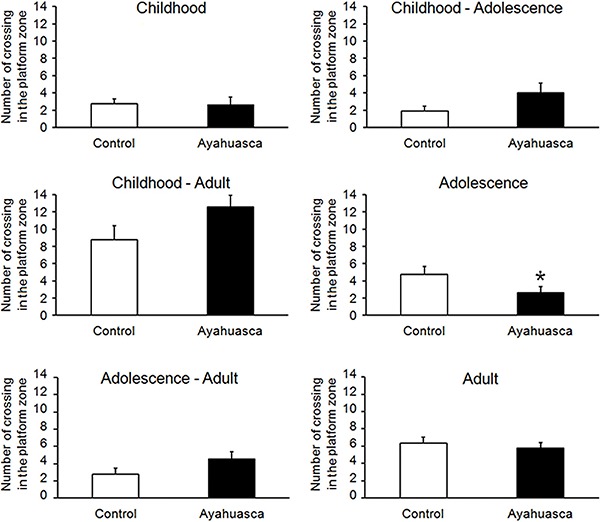
Number of times the platform zone was crossed during the probe trial session. Control mice received water whereas treated mice received ayahuasca (1.5 mL/kg, orally, in both cases) in the different periods of development. Data are reported as the mean±SE (n=8-12/group). *P<0.05 compared to the corresponding control group (Student's unpaired *t*-test).

*Spatial reference memory retrieval*. The time spent in the platform quadrant and the number of times the platform zone was crossed during the probe trial session are shown in Figures 5 and 6, respectively. In relation to the time spent in the platform quadrant, Student's *t*-test detected a significant effect of treatment in adolescence, with mice that received ayahuasca spending less time in the platform quadrant than the control group [t(20)=-2.6937; P=0.0139]. There was no significant effect of treatment in the childhood [t(19)=0.1904; P=0.8509], adult [t(21)=-1.5820; P=0.1285], childhood-adolescence [t(15)=0.6299; P=0.5381], childhood-adult [t(14)=0.6034; P=0.5558] and adolescent-adult [t(17)=-0.2871; P=0.7774] groups. Student's *t*-test also detected a significant effect of treatment on the number of times the platform quadrant was crossed in adolescence, with ayahuasca-treated mice crossing the quadrant fewer times than the control group [t(20)=-2.3350; P=0.0300]. There was no significant effect of treatment in the childhood [t(19)=-0.6269; P=0.7393], adult [t(21)=-0.4154; P=0.6820], childhood-adolescence [t(15)=0.1805; P=0.8591], childhood-adult [t(14)=-0.6853; P=0.5042] and adolescent-adult [t(17)=-0.0989; P=0.9223] groups.

## Discussion

Previous experimental investigations of the behavioral effects of ayahuasca used acute or chronic administration protocols that did not reflect the use pattern of this beverage in humans (35–37). The distinguishing feature of our experimental protocol was the chronic intermittent exposure to ayahuasca that mimicked the use in a ritualistic context. While it is well established that emotional and cognitive changes induced by ayahuasca are attributable primarily to the activation of 5-HT_1A_ and 5-HT_2_ receptors (3–6), in the present study the mice were submitted to behavioral tests without the acute pharmacological effects of ayahuasca. Therefore, the behavioral changes observed here probably reflected neural plasticity induced by chronic intermittent exposure to ayahuasca rather than the acute stimulation of these receptors induced by a single ayahuasca treatment.

Our results indicated that intermittent chronic exposure to ayahuasca caused anxiety and memory impairment in childhood and adolescence, respectively. However, these behavioral changes were not long-lasting since there was no anxiety in the childhood-adolescence and childhood-adult groups, and no memory impairment in the adolescence-adult group.

A double-blind placebo-controlled clinical study in which adult subjects ingested ayahuasca for at least 10 consecutive years showed that, while under the acute effects of ayahuasca, participants scored lower on the scales for panic and hopelessness-related states, but showed no change in state- or trait-anxiety (29). In contrast, a psychiatric assessment found that while adolescent users of ayahuasca within a religious context had lower positive scores for anxiety than the control group, this difference was not significant (15). These clinical findings suggest that age is a factor that could influence the modulatory effects of ayahuasca on anxiety.

In relation to experimental studies, Favaro et al. (36) showed that long-term ayahuasca administration did not change the anxiety parameters of adult rats in the EPM test. Our findings agree with this study since ayahuasca did not alter the open arms exploration, regardless of the experimental group. Open arms exploration (entries into and/or the time spent in the arms) is the most commonly used parameter to evaluate an anxiety-like state in the EPM test. Although the predictive validity of the open arms parameters has been established for benzodiazepine-related drugs, far less has been reported for serotonergic drugs. Additional evaluation of defensive patterns, such as risk assessment, improves the accuracy of the EPM test, including the assessment of serotonergic-related drugs (31–33,38)>. In this regard, although the psychiatric and neuropsychological parameters evaluated in adolescents exposed to ayahuasca have not shown significant alterations, even with intrauterine exposure, few of these studies have examined the effects in childhood (15,16). As shown here, treating mice with ayahuasca increased risk assessment behavior in childhood but not in adolescence or adulthood, suggesting that childhood is a sensitive period for the anxiety-like effects of ayahuasca. However, the effects of ayahuasca exposure during childhood do not persist into later stages. Therefore, our results suggest that age can be an important factor in determining the emotional effects of ayahuasca, even if transient.

With regard to cognitive aspects, a recent study (39) showed a cortical thinning in the posterior cingulated cortex of ayahuasca users. In the same study, correlational analysis showed that the cortical thinning was proportional to the intensity and duration of previous ayahuasca consumption. This structural change could potentially affect attentional processes, self-referential thought and internal mentation (23). However, three other studies found no evidence of impaired executive functions in adolescent (16) and adult (23,28) ayahuasca users. One study investigated the effects of acute ayahuasca exposure on the neuropsychological performance of long-term experienced and occasional ayahuasca users, using the Stroop, Sternberg, and Tower of London tasks prior to and following ayahuasca intake. Ayahuasca impaired the working memory in both groups. However, detrimental effects on higher cognition were only observed in occasional ayahuasca users, suggesting that compensatory or neuromodulatory effects associated with long-term ayahuasca intake could underlie the preservation of executive functions in long-term experienced users (28).

Another study done in regular ayahuasca users (n=127) and controls (who were participating in non-ayahuasca religions; n=115) evaluated at baseline and one year later found that ayahuasca users showed better performance in the Stroop test, the Wisconsin Card Sorting test and the Letter-Number Sequencing task from the WAIS-III, and better scores on the Frontal Systems Behavior Scale; this profile was maintained one year later (23).

Finally, neuropsychological tests in adolescents who used ayahuasca in a religious setting showed that their performances were similar to those of a control group of adolescents who did not use ayahuasca; the parameters evaluated included speeded attention, visual searching, sequencing, psychomotor speed, verbal and visual abilities, memory, and mental flexibility (16). However, lower scores for the ayahuasca group were observed in trials II and IV and in the total score of trials I to IV of the WHO/UCLA Auditory Verbal Learning test. Nevertheless, the mean raw scores of trials II and IV of both groups did not differ significantly from adolescent normative data.

Ayahuasca alters the serotonin levels in temporal lobe structures of rats (40). Surprisingly, only one study in rodents has investigated the effects of ayahuasca on learning and memory related to the temporal lobe (36). In that study, adult rats were treated chronically (daily oral gavage for 30 days) with a freeze-dried ayahuasca extract at doses of 120, 240, and 480 mg/kg. Treatment with ayahuasca changed the contextual association of emotional events, but there was no evidence of memory impairment in the MWM test (a spatial reference memory-dependent model) or of tone fear conditioning, although the dose of 120 mg/kg increased contextual conditioned fear (36). In the present study, although all of the experimental groups learned the task, adolescent rats spent less time in the platform quadrant during the probe trial, which suggested spatial reference memory impairment. This finding indicated that the effect of ayahuasca on hippocampal-dependent memory tasks was at least partly influenced by the period of life in which exposure to ayahuasca occurs.

In countries of the Amazon Basin such as Brazil, Ecuador, and Peru, the use of ayahuasca is fundamentally legal, both in the indigenous context and among non-indigenous religious groups (11). The ritual use of ayahuasca in religious contexts often starts during childhood or early adolescence, and may continue into adulthood and throughout life (20). In this study, we sought to investigate the possible ontogenic effects of ayahuasca through chronic intermittent treatment (twice a week) that mimicked the ritualistic use of the beverage. Our results showed that this experimental protocol promoted anxiety or memory impairment when consumption occurred in childhood or adolescence, respectively. These behavioral changes were not long-lasting because they were not observed in adult groups. Since ayahuasca use in religious groups may occur in similar age groups to those studied here in mice, it would seem worthwhile to examine the potential influence of such consumption on the corresponding parameters in humans.
